# In Memoriam: Professor Philippe Jeandet – an outstanding scientist and his legacy in natural product chemistry and bioactivity

**DOI:** 10.5114/bta/208879

**Published:** 2025-09-22

**Authors:** Iwona Morkunas, Magda Formela-Luboińska, Side Selin Su Yirmibesoglu, Jan Bocianowski, Waldemar Bednarski, Jacek Kęsy, Paulina Glazińska, Agnieszka Woźniak, Van Chung Mai, Mehmet Zafer Dogu, Anielkis Batista, Dorota Narożna, Renata Rucińska-Sobkowiak, Mateusz Labudda, Ebru Kafkas, Salih Kafkas, Aziz Aziz, Patricia Trotel-Aziz, Sylvain Cordelier, Cédric Jacquard, Christophe Clément, Chandra Mohan, Michał Tomczyk, Eduardo Sobarzo-Sánchez, Roque Bru, Ascension Martínez-Márquez, Gaber El-Saber Batiha, Mattheos Koffas, Alessandro Vannozzi, Md. Sahab Uddin, Seyed Mohammad Nabavi, Maurizio Battino, Adrián Matencio, Francesco Trotta, Haroon Khan

**Affiliations:** 1 Department of Plant Physiology, Faculty of Agriculture, Horticulture and Biotechnology, Poznań University of Life Sciences, Poznań, Poland; 2 Molecular Biology and Genetics Section, Department of Biology, Faculty of Science, Ege University, Bornova, Izmir, Turkey; 3 Department of Mathematical and Statistical Methods, Faculty of Agriculture, Horticulture and Biotechnology, Poznań University of Life Sciences, Poznań, Poland; 4 Institute of Molecular Physics, Polish Academy of Sciences, Poznań, Poland; 5 Department of Plant Physiology and Biotechnology, Faculty of Biological and Veterinary Sciences, Nicolaus Copernicus University in Toruń, Poland; 6 Vinh University, Nghe An Province, Vietnam; 7 Polytechnic Institute of Huila, Universidade Mandume ya Ndemufayo, Lubango, Angola; 8 Department of Biochemistry and Biotechnology, Faculty of Agriculture, Horticulture and Biotechnology, Poznań University of Life Sciences, Poznań, Poland; 9 Department of Plant Ecophysiology, Faculty of Biology, Adam Mickiewicz University, Poznań, Poland; 10 Department of Biochemistry and Microbiology, Institute of Biology, Warsaw University of Life Sciences – SGGW, Warsaw, Poland; 11 Department of Horticulture, University of Çukurova, Adana, Turkey; 12 University of Reims Champagne-Ardenne, INRAE, RIBP, USC 1488, ‘Induced Resistance and Plant Bioprotection’, Reims, France; 13 Department of Chemistry, School of Basic and Applied Sciences, K.R. Mangalam University, Gurugram, India; 14 Department of Biology and Pharmacognosy, Faculty of Pharmacy with the Division of Laboratory Medicine, Medical University of Bialystok, Poland; 15 Centro de Investigación en Ingeniería de Materiales-CIIMAT, Facultad de Medicina y Ciencias de la Salud, Universidad Central de Chile, Santiago, Chile; 16 Plant Proteomics and Functional Genomics Group, Department of Biochemistry and Molecular Biology and Soil Science and Agricultural Chemistry, Faculty of Science, University of Alicante, Spain; 17 Department of Pharmacology and Toxicology, Faculty of Veterinary Medicine, Damanhour University, AlBeheira, Egypt; 18 Dorothy and Fred Chau ‘71 Constellation Professor, Center for Biotechnology and Interdisciplinary Studies, Rensselaer Polytechnic Institute, Troy, NY, USA; 19 Laboratory of Genetics and Genomics for Breeding, Department of Agronomy, Food, Natural Resources, Animals and Environment, University of Padova, Campus of Agripolis – V.le dell’Universitŕ 16, Legnaro (PD), Italy; 20 Department of Pharmacy, Southeast University, Dhaka, Bangladesh; 21 Pharmakon Neuroscience Research Network, Dhaka, Bangladesh; 22 Advanced Medical Pharma (AMP-Biotec), Biopharmaceutical Innovation Centre, Via Cortenocera, San Salvatore Telesino, BN, Italy; 23 Department of Clinical Sciences, Faculty of Medicine, Polytechnic University of Marche, Ancona, Italy; 24 Research Group on Food, Nutritional Biochemistry and Health, Universidad Europea del Atlántico, Santander, Spain; 25 Departamento de Bioquímica y Biología Molecular-A, Facultad de Biología, Universidad de Murcia-Regional Campus of International Excellence “Campus Mare Nostrum”, Murcia, Spain; 26 Department of Chemistry, University of Turin, Italy; 27 Department of Pharmacy, Faculty of Chemical and Life Sciences, Abdul Wali Khan University Mardan, Pakistan; 28 Department of Pharmacy, Korea University, Sejong, South Korea

**Keywords:** chemical structure of natural products and bioactivity stilbenoids, resveratrol and derivatives, wine microbiology, the use of stilbenes in medical research, hormesis phenomenon, sugar signaling, defense responses to biotic stress

## Abstract

Professor Philippe Jeandet was one of the world’s leading biologists and plant biochemists, best known for his research on the chemical structure of natural products and their bioactivity, particularly that of stilbenoids. His scientific interests primarily focused on resveratrol (trans-3,5,4'-trihydroxystilbene), a stilbene with a wide range of biological activities. Additionally, his work highlighted the potential of combining pharmacological treatments with the use of natural products of plant origin, which have made significant contributions to the treatment of various diseases. He leaves behind a legacy of groundbreaking research and a lasting influence in the field. He was also involved in research on sugar signaling during plant responses to abiotic and biotic stress factors, as well as the role of signaling molecules in fruit development. His scientific achievements demonstrate that he was, first and foremost, a dedicated scientist – but also a honourable colleague who understood and respected the work of others.

Prof. Dr. Philippe Jeandet from the University of Reims Champagne-Ardenne in France was a remarkable professor whose scientific achievements deserve the highest recognition. He belonged to the foremost scientists in biology and biochemistry, both in France and globally. Notably, Prof. Philippe Jeandet was ranked among the top 1% of the most influential researchers worldwide, as published by Stanford University, based on data from the Scopus (Elsevier) platform. It is admirable that he held a Discipline-specific h-index of 69, with 17253 citations to his name. He was the author or co-author of more than 300 articles in the fields of biology, biochemistry, phytopathology, molecular biology, and medicine. Over 200 of these articles were published in journals indexed in the Journal Citation Reports.

His outstanding abilities, scientific passion, and determination – evident from the beginning of his career – are also reflected in his academic accomplishments. He earned two doctoral degrees from the University of Burgundy, one of the top-ranked institutions in several leading international rankings. His first PhD was in Plant Physiology and Biochemistry, completed in 1991, followed by a second PhD in Plant Pathology and Biochemistry in 1996. Jeandet also served as a visiting professor at the Swiss Federal Agronomic Station in Changins (CH) in 1994 and at the University of Kentucky (USA) in 1997. That same year, he was appointed Full Professor at the University of Reims, and in 2010, he was named Professor of High Distinction (Exceptional Class), continuing his service at the University of Reims. It is worth noting that his research was primarily conducted at the University of Burgundy (his alma mater) and the University of Reims.

Throughout his career, beginning in 1995, he received the Research Excellence Award from the French Ministry of Research and Higher Education five times – a testament not only to his talent but also to his tireless dedication. He was always guided by the belief that one must learn and work hard to publish at a high level and with impact. Prof. Philippe Jeandet was also known for his collegiality toward other scientists. He knew how to appreciate the work of others while remaining both modest and demanding of himself.

His entire scientific achievements definitively demonstrates that he was, first and foremost, a scientist – although he also held several important positions throughout his professional career. Prof. Philippe Jeandet served successively as Director of the Laboratory of Enology and Applied Chemistry at the Faculty of Science in Reims, Director of the Vine and Wine of Champagne Research Unit, and Deputy Director of Research and Technology in the Champagne-Ardenne Region. He contributed to the creation of the Georges Chappaz Institute of Vine and Wine in Champagne and was a member of its board of directors. In line with his scientific interests, he also co-directed a research team on resveratrol at the Induced Resistance and Plant Bioprotection Laboratory at the University of Reims.

The primary focus of Prof. Philippe Jeandet’s research was the chemical structure of natural products and their bioactivity, particularly that of stilbenoids. From the beginning of his professional career to his most recent work, his scientific interests remained centered on resveratrol (trans-3,5,4'-trihydroxystilbene) – the most extensively studied stilbene, known for its wide spectrum of biological activity. This secondary metabolite, synthesized via the phenylpropanoid pathway, continues to attract special attention from the scientific community. As a grapevine phytoalexin, resveratrol not only contributes to plant defense against abiotic and biotic stressors but also holds promise as a potential pharmaceutical agent due to its beneficial effects on human health (e.g., antitumor, antioxidant, anti-inflammatory, cardioprotective, neuroprotective, and antidiabetic actions).

Numerous publications by Prof. Philippe Jeandet have reported that stilbenoids, including resveratrol, may offer therapeutic potential in various diseases, as they can modulate the activity of several key signaling pathways – such as phosphatidylinositol 3′-kinase (PI3K)/Akt and nuclear factor-kappa B (NF-κB).

At the beginning of Philippe Jeandet’s scientific career, his first publication addressed his leading research topic in an article entitled “Une réflexion sur les mécanismes morphologiques et biochimiques de l’interaction vigne–*Botrytis*” (translated as “Account on Morphological and Biochemical Mechanisms of Vine– *Botrytis* Interaction [*Botrytis cinerea*]”), published in *Bulletin de l’OIV* in France in 1989 (Jeandet and Bessis 1989). In this early work, he focused on the chemical composition of the grape skin and the biological role of stilbene phytoalexins – among the most important secondary metabolites involved in the vine’s resistance to fungal pathogens such as *B. cinerea*.

His first significant experimental article, co-authored with Prof. Roger Bessis – his PhD thesis supervisor – focused on resveratrol, a phytoalexin from the *Vitaceae* family produced in response to fungal attacks or physical injury. This study, titled “The Production of Resveratrol (3,5,4′-trihydroxystilbene) by Grape Berries in Different Developmental Stages,” was published in the *American Journal of Enology and Viticulture* in 1991 (Jeandet et al. 1991). In this publication, Philippe Jeandet identified the localization of resveratrol in grape berries of *Vitis vinifera* L. and *V. labrusca* – an important finding in the context of plant defense mechanisms against fungal pathogens. Research results of Philippe Jeandet also showed that resveratrol biosynthesis occurs in different developmental stages of berries under UV-irradiation. Another key result was the observed negative correlation between resveratrol content in grape skins and sugar levels in the berries during ripening.

Subsequently, Philippe Jeandet published a study analyzing resveratrol in Burgundy wines – Pinot Noir, Chardonnay, and Aligoté – using gas chromatography (GC) and gas chromatography-mass spectrometry (GC-MS) techniques (Jeandet et al. 1993). This work highlighted GC and GC-MS as practical tools for the determination of resveratrol in wines. The analysis revealed significantly higher concentrations of resveratrol in red wines compared to those made from white grape varieties.

Among his particularly noteworthy contributions was a 1995 article in the *Journal of Agricultural and Food Chemistry* titled “Effect of Enological Practices on the Resveratrol Isomer Content of Wine” (Jeandet et al. 1995). In this study, Jeandet and his co-authors reported that two factors can modify resveratrol levels in wine, i.e., the influence of classical white or red winemaking practices and the effect of *B. cinerea* grape infection on the resveratrol content of wines. The first valuable result of this experimental work was to show that lower concentrations of resveratrol were present in wines made from highly *Botrytis*-infected grapes than in wines from healthy and moderately infected grapes. A second key result was the clear identification of high quantities of the *cis*-isomer of resveratrol in wine, despite this isomer being only minimally detectable in fresh grapes.

In the years that followed, Philippe Jeandet continued to expand his research on resveratrol. His work encompassed not only biosynthesis, metabolism, molecular engineering, and the biological functions of resveratrol and other stilbene phytoalexins in plant material, but also the fungitoxic activity of resveratrol. A major achievement in this area was his demonstration that resveratrol inhibits both the mycelial growth and the germination of *B. cinerea* conidia. These findings, based on *in vitro* experiments and light microscopy observations, were published in the *Journal of Chemical Ecology* in 1997 (Adrian et al. 1997).

In addition to his scientific publications, Philippe Jeandet was the first to demonstrate that certain metallic salts, such as aluminum chloride, could act as powerful inducers of resveratrol production in grapevine leaves (patent A01N 59/06, “Use of aluminum chloride as a resveratrol synthesis elicitor” 29 May 1997).

In his scientific journey, Prof. Jeandet has not only developed scientific research techniques, which contributed to producing interesting research results on resveratrol and stilbene derivatives, but he has also written many interesting literature reviews, thus contributing to deepening the knowledge of the scientific community on stilbene derivatives. In the present Editorial, we focused on articles of Prof. Philippe Jeandet, which have attracted particular attention from the scientific community due to the high number of citations. One of his notable review articles is titled “Phytoalexins from the Vitaceae: Biosynthesis, Phytoalexin Gene Expression in Transgenic Plants, Antifungal Activity, and Metabolism,” published in the *Journal of Agricultural and Food Chemistry* in 2002 (Jeandet et al. 2002). Another key contribution is the article “Bioproduction of resveratrol and stilbene derivatives by plant cells and microorganisms”, published in *Trends in Biotechnology* in 2009 (Donnez et al. 2009). A third influential review, “Biosynthesis, metabolism, molecular engineering, and biological functions of stilbene phytoalexins in plants”, was published in *BioFactors* in 2010 (Jeandet et al. 2010).

In these comprehensive reviews, Prof. Jeandet examined the biosynthesis of stilbene phytoalexins – especially in grapevine (*Vitis* spp.) – as well as the synthesis and chemical analysis of phytoalexins (resveratrol and its derivatives), their induction by *B. cinerea*, and their biological activity. He also elucidated the role of stilbene synthase (STS), the key enzyme that catalyzes the formation of the stilbene skeleton in a single reaction from malonyl-CoA and *p*-coumaroyl-CoA to produce *trans*-resveratrol.

Prof. Jeandet and his co-authors reported that in *V. vinifera* L. – one of the richest natural sources of resveratrol – genome sequencing revealed high expression of STS genes. These genes form a multigene family, suggesting the importance of stilbene metabolism in this species. Each of these review papers contributed new insights into the ongoing progress of resveratrol research. For example, they emphasized STS gene transfer experiments in plants and the synthesis of foreign phytoalexins – specifically resveratrol and its glucosides – which conferred enhanced resistance to various pathogens in genetically modified plants.

Jeandet’s exceptional scientific activity also led to his appointment as Guest Editor of the journal *Molecules* (MDPI, Basel, Switzerland) in 2015, where he supervised the Special Issue titled “Phytoalexins: Current Progress and Future Prospects.”

Prof. Philippe Jeandet and his colleagues was publication of the two review papers on stilbenes in *Natural Products Reports* (IF 15.111) and in *Biotechnology Advances* (IF 17.681), in 2021. The mentioned publications are characterized by high citation rates. The first work, entitled “Phytostilbenes as agrochemicals: biosynthesis, bioactivity, metabolic engineering and biotechnology”, has been cited 99 times to date. This comprehensive review covers stilbene chemistry and biochemistry, regulation of their biosynthesis, biological roles in plants, molecular engineering of stilbene pathways in both plants and microbes, and their biotechnological production using plant cell systems (Jeandet et al. 2021a).

The second paper, published in *Biotechnology Advances* (IF 17.681), has been cited 44 times since 2021. It explored the role of cyclodextrins in stilbene chemistry at the physico-chemical, biomedical, and biotechnological levels (Jeandet et al. 2021b). Prof. Jeandet and his collaborators addressed the induction of resveratrol production by cyclodextrins, the synergistic effects of combining cyclodextrins with methyl jasmonate in plant cell systems, and the biosynthesis of stilbenes in the presence of cyclodextrins. In turn, a synthetic and critical analysis of the methods used for the production of high molecular-ordered stilbene oligomers of potential biomedical interest, data regarding the approaches employed to prepare them by total synthesis, use of biomimetic approaches or through plant systems, were presented in a review published in *Natural Product Reports*, in 2023 (Jeandet et al. 2023a).

Another 2023 publication, titled “Use of Elicitors and Beneficial Bacteria to Induce and Prime the Stilbene Phytoalexin Response: Main Experiments and Applications to Grapevine Disease Resistance” (Jeandet et al. 2023b), aimed to assess whether inducing stilbene phytoalexin production could protect plants against pathogens. The review discussed the potential role of grapevine phytoalexins exhibiting biocidal activity against a wide range of plant pathogens.

It is also worth mentioning that Prof. Philippe Jeandet’s second major research focus was wine microbiology. Within this area, he and his co-workers investigated the ability of yeast to remove thiols in both synthetic media and wine (Vasserot et al. 2003). Moreover, they proposed a novel hypothesis suggesting that acetic acid could alter yeast metabolism by reducing the activity of the NADP^+^-dependent aldehyde dehydrogenase Ald6p. This work was published in *Food Chemistry* (Vasserot et al. 2010).

Another important study led by Prof. Jeandet addressed an alternative method for preventing wine haze formation (Younes et al. 2013). In this research, *Saccharomyces cerevisiae* PlR1 – a strain isolated from *Pinot noir* grapes in the Champagne region – was shown to secrete acid proteolytic enzymes capable of degrading bovine serum albumin. The results provided evidence that the hydrolyzed proteins corresponded to pathogenesis-related proteins, particularly thaumatin-like proteins (TL) and chitinases, which are likely involved in the formation of wine haze.

The third major research area of Prof. Jeandet focused on physicochemistry as applied to wine. This included the physics of bubbles in champagne, the physico-chemistry of foaming properties in champagne and sparkling wine, studies on grape and wine proteins, applications of spectroscopic methods to enology, and the use of proteomics and metabolomics in grapevine and wine research.

In this field, Prof. Philippe Jeandet co-authored several articles investigating the phenomenon of champagne bubbling. His research included close observation of the three main stages in a champagne bubble’s life cycle: bubble nucleation, bubble ascent, and the collapse of the bubble at the liquid’s surface. These findings were published in the article “Effervescence in a Glass of Champagne: A Bubble Story,” featured in *Europhysics News* in 2002 (Liger-Belair and Jeandet 2002). The topic was also explored in a conference article titled “Deterministic Process of the Transitions between Different Bubbling Regimes of Some Nucleation Sites in Champagne and Sparkling Wines”, published in *Macromolecules and Secondary Metabolites of Grapevine and Wines* in 2007 (Liger-Belair et al. 2007), which described the phenomenon of bubbling transitions over time during gas discharge.

It is also worth noting that Prof. Philippe Jeandet was the first author of an experimental article titled “Control of Oxygen Enrichment during Bottling in the Sparkling Winemaking Process”, published in *Food* in 2008 (Jeandet et al. 2008). In this study, he evaluated the benefits of a new bottling apparatus designed to control the oxygen environment in the neck of sparkling wine bottles. He highlighted the importance of the impact of oxygen management before bottling on wine organoleptic properties. Moreover, he co-authored a critical review, “Recent advances in the science of champagne bubbles”, that summarized recent advances obtained during the past decade concerning the physicochemical processes behind the nucleation, rise, and burst of bubbles found in glasses poured with champagne and sparkling wines (Liger-Belair et al. 2008). This critical review was published in *Chemical Society Reviews* in 2008. Prof. Jeandet also had a strong interest in the study of must and wine proteins, particularly their physicochemical properties. Within this research framework, he worked on a broad range of topics, including the purification and quantification of proteins in grape juice, must, and wine – such as vacuolar grape invertase and pathogenesis-related (PR) proteins, a lectin fraction from *Chardonnay* grape juice, lysozyme in *Pinot noir* and *Chardonnay* Champagne wines, and chitinase and thaumatin-like proteins in grape juices and wines. He also investigated two pectinolytic enzymes secreted by *B. cinerea* in botrytized wine.

To conduct these studies, Prof. Jeandet employed various analytical techniques, including electrophoresis (SDS-PAGE and 2D-GE), chromatography (fast protein liquid chromatography (FPLC), high-performance liquid chromatography (HPLC), ultra-HPLC (UHPLC), nanoLC-chip-MS/MS), proteomics, and immunological methods such as enzyme-linked immunosorbent assay (ELISA).

Additionally, he investigated the origin of proteins in *Chardonnay* wines to better understand the fermentation process and foam stabilization in Champagne. His research demonstrated that proteins, despite their low concentrations, play a significant role in foam stabilization in Champagne base wines (Dambrouck et al. 2003). Prof. Philippe Jeandet and co-workers, using various polyclonal antibodies, demonstrated that most of the wine proteins came from grapes and many of them were glycoproteins. Some proteins were also found to originate from yeast.

Moreover, Prof. Jeandet demonstrated that infection by *B. cinerea*, the causal agent of gray mold, negatively affects the foaming properties of Champagne base wines. This effect is due to the plant’s immune responses to the pathogen, which result in the degradation, repression, or differential expression of grape proteins during infection.

Following the identification and characterization of protein structures in wine and Champagne, his research progressed to investigating the removal of endogenous proteins. This included studies on the use of lysozyme additions to musts before and after treatments with bentonite or charcoal, as well as the clarification of *Muscat* must treated with pectinase.

Prof. Philippe Jeandet also conducted research on alternative fining agents for wine, including enzymatically hydrolyzed glutens (EHG), casein, gelatin–tannin associations, fish glue, bentonite, hydrolyzed and deamidated wheat proteins, vital gluten, and novel enological bentonites such as polylysine- and polyglutamic acid-interacted synthetic montmorillonite. Two natural bentonites from Greece and Turkey were characterized using X-ray diffraction, thermal analysis, and solid-state nuclear magnetic resonance (NMR) spectroscopy – both before and after Na_2_CO_3_ activation. Additionally, natural montmorillonite clay (Mt) absorbed with β-lactoglobulin (BLG) was studied using zetametry, X-ray diffraction, transmission electron microscopy, fluorescence spectroscopy, and solid-state NMR.

The results of this research were published in *Solid State Nuclear Magnetic Resonance* in 2006 (Gougeon et al. 2006). It is also worth emphasizing the fact that a great achievement of Prof. Philippe Jeandet and his co-workers was the study of the 170-year-old Champagne from the bottom of the Baltic Sea. Prof. Philippe Jeandet and co-workers performed a multiplatform analytical investigation of 170-year-old Champagne bottles found in a shipwreck on the bottom of the Baltic Sea. Information on this topic appeared in *Nature* (Wilkinson 2015). Bottles of champagne were discovered in the Finnish Aland archipelago, and identified as early nineteenth-century by engravings in their corks. The detailed scientific findings were presented in the paper titled “Chemical Messages in 170-year-old Champagne Bottles from the Baltic Sea: Revealing Tastes from the Past”, published in the *Proceedings of the National Academy of Sciences* in 2015 (Jeandet et al. 2015).

Prof. Philippe Jeandet’s extensive research interests were reflected in numerous scientific publications, distinguished by their interdisciplinary approach to the subjects analyzed. Notably, he was a co-author of many works addressing broad issues in the field of medical sciences.

Among his significant achievements are studies on the therapeutic potential of polyphenols – such as epigallocatechin-3-gallate, curcumin, resveratrol, quercetin, and methylated polyphenols like berberine – in the treatment of neurodegenerative diseases (NDs), including Parkinson’s and Alzheimer’s disease. These include the articles “Neuroprotective Role of Polyphenols against Oxidative Stress-mediated Neurodegeneration” and “Neuroinflammatory Signaling in the Pathogenesis of Alzheimer’s Disease,” published in 2020 and 2022, respectively (Uddin et al. 2020; Uddin et al. 2022). In these studies, Prof. Jeandet and his collaborators demonstrated that polyphenols offer protective effects against neuronal damage.

More recently, his research also explored the role of various phytochemicals in the prevention and treatment of different types of cancer, with a particular focus on ginsenosides. Prof. Jeandet and his colleagues investigated the potential of combining natural products with chemotherapeutic agents for cancer chemoprevention. This work culminated in the review article “Ginsenosides in Cancer: Targeting Cell Cycle Arrest and Apoptosis,” published in 2023 (Shah et al. 2023). The paper highlighted the anticancer properties of ginsenosides and detailed the mechanisms underlying their activity, supporting their potential application in novel anticancer strategies. Another extremely interesting paper entitled “Oral Microbiota in Cancer: Could the Bad Guy Turn Good with Application of Polyphenols?,” published in *Expert Reviews in Molecular Medicine* in 2023, explored the role of polyphenols in maintaining oral microbiome health and preventing disease, with a focus on conditions associated with oral dysbiosis (Antoniraj et al. 2023). The review analyzed existing literature showing that disruptions in the oral microbiota may contribute to the development and progression of several cancers, including oral, gastric, and pancreatic cancers.

Additionally, polydatin, or 3-O-β-d-resveratrol-glucopyranoside (PD), a stilbenoid compound found in knotweed (Polygonaceae), also drew the interest of Prof. Philippe Jeandet. In the review article “Uncovering the Anticancer Potential of Polydatin: A Mechanistic Insight,” published in *Molecules* in 2022 (Shah et al. 2022), he and his co-authors reported that polydatin possesses strong potential to modulate multiple signaling pathways involved in cancer development. The coauthorship of Prof. Philippe Jeandet, as an outstanding researcher in the biochemistry focusing on stilbenes, and especially on resveratrol in plants and plant products, in many review papers on the use of stilbenes in medical research demonstrated his broad interests, but also highlighted the potential of combining pharmacological treatment with the use of natural products of plant origin that display a large spectrum of biological activity.

A significant achievement of Prof. Philippe Jeandet and his co-workers was their research on the interaction of cationic and anionic dyes with clay minerals and organoclays. The colored solid materials obtained from this work were developed into value-added products – clay-based nanopigments – offering an environmentally friendly solution with no generation of secondary waste (Mohan et al. 2023).

In addition to his independent research, Prof. Philippe Jeandet was actively involved in scientific collaboration with a Polish research team. This partnership began in 2014 when he served as Guest Editor for *Molecules*, a leading international open-access chemistry journal. During his tenure, he invited Polish scientists to contribute to two Special Issues aligned with their research interests: “Phytoalexins: Current Progress and Future Prospects” in 2014 and “Structure, Chemical Analysis, Biosynthesis, Metabolism, Molecular Engineering and Biological Functions of Phytoalexins” in 2017. As part of these Special Issues, the Polish research team published two experimental articles: “Effects of Endogenous Signals and *Fusarium oxysporum* on the Mechanism Regulating Genistein Synthesis and Accumulation in Yellow Lupine and their Impact on Plant Cell Cytoskeleton” (Formela et al. 2014), and “The Influence of Lead on Generation of Signaling Molecules and Accumulation of Flavonoids in Pea Seedlings in Response to Pea Aphid Infestation” (Woźniak et al. 2017).

In the following years, Prof. Philippe Jeandet joined the research efforts of the Polish team. Their first coauthored publication, titled “The Role of Heavy Metals in Plant Response to Biotic Stress,” was published in *Molecules* in 2018 (Morkunas et al. 2018). This review paper focused on recent advances in research related to hormetic responses in plants, insects, and fungi. Prof. Jeandet and his colleagues demonstrated that the phenomenon of hormesis – where low doses of stressors elicit beneficial effects – can be observed across various organisms exposed to chemical, physical, or biological stressors.

The paper presented data on the effects of low (hormetic) and high (toxic) doses of heavy metals on plant defense responses, as well as the interactions between heavy metals and biotic stressors, such as insects and pathogenic fungi. It also addressed the complex relationships between environmental factors – including heavy metals – and invertebrates under natural, uncontrolled conditions.

In continuation of this research theme, Prof. Philippe Jeandet co-authored two experimental studies on hormesis. The first titled “The Influence of Lead and *Acyrthosiphon pisum* (Harris) on Generation of *Pisum sativum* Defense Signaling Molecules and Expression of Genes Involved in their Biosynthesis” (Woźniak et al. 2023), while the second is “The Effects of Lead and Cross-Talk between Lead and Pea Aphids on Defense Responses of Pea Seedlings” (Morkunas et al. 2024). Both publications were published in the *International Journal of Molecular Sciences* (IJMS). These studies, for the first time, demonstrated a significant induction in the expression of genes encoding enzymes involved in the biosynthesis of defense-related phytohormones in edible pea upon exposure of the plant to varying concentrations of lead, with the low concentration potentially leading to the hormesis effect and the high concentration causing a sublethal effect as well as during infestation by phytophagous pea aphid (Woźniak et al. 2023). In turn, the greatest novelty of the second experimental article was to show for the first time the involvement of sucrose, glucose, and invertases in the defense response of the edible pea on the exposure of pea seedlings to varying concentrations of lead. Additionally, Prof. Philippe Jeandet and colleagues showed for the first time, changes in the expressions of *isoflavone synthase* (IFS) and *6*α-*hydroxymaackiain 3-O-methyltransferase* (*HMM*) genes in pea seedlings subjected to both hormetic and sublethal doses of lead, as well as during the cross-talk between lead exposure and *A. pisum* infestation (Morkunas et al. 2024).

Moreover, Prof. Philippe Jeandet was also involved in the second major research topic of the Polish scientific team, focusing on the regulatory role of sugars in plant responses to biotic stress. Together with Polish scientists, he served as Editor of the Special Issue titled “The Role of Sugars in Plant Responses to Stress and Their Regulatory Function During Development,” in the IJMS from 2020 to 2025.

It is also worth highlighting that Prof. Philippe Jeandet co-authored two experimental publications within the scope of this research, both addressing the role of soluble sugars in the defense response to fungal pathogens. First original article entitled “The Role of Sugars in the Regulation of the Level of Endogenous Signaling Molecules during Defense Response of Yellow Lupine to *Fusarium oxysporum*” (Formela-Luboińska et al. 2020a), and the second, “The Role of Saccharides in the Mechanisms of Pathogenicity of *Fusarium oxysporum* f. sp. *lupini* in Yellow Lupine (*Lupinus luteus* L.)” (Formela-Luboińska et al. 2020b), were both published in IJMS in 2020. In the first article (Formela-Luboińska et al. 2020a), the authors revealed for the first time the involvement of soluble sugars – such as sucrose, glucose, and fructose – as primary signaling molecules regulating the levels of defense-related phytohormones and hydrogen peroxide during the response of *L. luteus* to the hemibiotrophic fungus *F. oxysporum* f. sp. *lupini*. Prof. Philippe Jeandet and co-workers showed a protective effect of soluble sugars in yellow lupine embryo axes in the restricted development of infection and fusariosis. In turn, in the second experimental paper, Prof. Philippe Jeandet and co-authors demonstrated the correlation between the pathogenicity of *F. oxysporum* f. sp. *lupini* and the concentration of soluble sugars in the yellow lupine embryo axes (Formela-Luboińska et al. 2020b). The study concluded that high levels of sucrose and monosaccharides may reduce the virulence of *F. oxysporum* and limit both infection development and fusariosis progression. This fruitful collaboration concluded in the publication of an Editorial article titled “The Role of Sugars in Plant Responses to Stress and Their Regulatory Function During Development” in the aforementioned Special Issue (Jeandet et al. 2022).

Additionally, Prof. Philippe Jeandet co-authored several research papers focused on fruit development, conducted as part of international collaborations. One line of research investigated the biochemical and physical properties of apple fruits cultivated in the Wielkopolska region of Poland. The results demonstrated that the apple varieties examined varied significantly in their biochemical composition and physical characteristics (Yoon et al. 2020). The study revealed differences in sugar profiles and concentrations, levels of semiquinone radicals, abscisic acid, and organic acids among the analyzed apple varieties.

Prof. Jeandet also contributed to research on the physiological, biochemical, and organoleptic changes during the ripening of strawberry fruit (*Fragaria × ananassa* Duch.), one of the most widely consumed fruits globally due to its high nutritional value and healthpromoting properties (Ayvaz Sönmez et al. 2021). This study demonstrated the involvement of phenylalanine ammonia-lyase (PAL), a key enzyme initiating phenylpropanoid metabolism and anthocyanin biosynthesis, in the fruits of two strawberry cultivars – “Rubygem” and “Fortuna,” – at different stages of ripening. The findings indicated that the longer the fruit ripened, the higher the activity of these enzymes since “Fortuna” and “Rubygem” exhibited significantly different PAL and invertase activity throughout the ripening phases (Ayvaz Sönmez et al. 2021). Moreover, Prof. Philippe Jeandet was a co-author of an experimental article entitled “Profile of Semiquinone Radicals, Phytohormones and Sugars in *Pistacia vera* L. cv. Kirmizi Development” (Morkunas et al. 2021).

Their findings revealed, for the first time, a significant increase in semiquinone radical production and fluctuations in paramagnetic metal ions (Mn^2+^) in the fruits of pistachio trees (*Pistacia vera* L. cv. Kirmizi) following flower cluster thinning during development. Additionally, Prof. Philippe Jeandet and his co-workers highlighted the crucial role of signaling molecules – particularly phytohormones and soluble sugars – in pistachio fruit development, especially during the early stages.

More recently, Prof. Jeandet co-authored a study investigating the role of silver (AgNPs) and selenium (SeNPs) nanoparticles, applied at different concentrations, in regulating plant defense responses to biotic stresses (Batista et al. 2025). The study demonstrated that nanoparticles – especially AgNPs and SeNPs – can be effectively used to treat infections and protect plants from phytopathogens, showing promise as tools in plant disease management. In addition to his research contributions, Prof. Philippe Jeandet served as Guest Editor of the Special Issue “Molecular Advances in Abiotic Stress Signaling in Plants: Focus on Atmospheric Stressors” in the IJMS in 2024 (Labudda and Jeandet 2025).

In addition to his outstanding publications, Prof. Philippe Jeandet also coordinated several national and international research projects. His scientific achievements include an international patent, over 270 communications presented at numerous symposia or congresses, the organization of international scientific meetings, and the creation of a dedicated symposium series titled “Macromolecules and Secondary Metabolites of Grapevine and Wine.” He also edited three books and curated 15 special issues in scientific journals. Moreover, he served on the editorial boards of 15 academic journals and acted as an occasional referee for 75 different scientific publications.

Many of us had the privilege of collaborating with Prof. Philippe Jeandet on various scientific projects. Of particular significance is the highly cited review published in *Biotechnology Advances*, which provides a comprehensive analysis of the metabolic engineering of flavonoid compounds, detailing their chemical structures, biochemical transformations, functions, and therapeutic applications (Nabavi et al. 2020). Many of our outstanding joint projects have not been completed despite being started, but we hope that we will be their worthy successors.

Professor Philippe Jeandet was not only an outstanding academic, driven by immense scientific passion and dedicated to collaboration with scientists from around the world, but also a remarkable friend – someone who deeply understood and respected his colleagues. As colleagues and collaborators of Prof. Philippe Jeandet, we wish to pay tribute to him once again and share this heartfelt message with the scientific community: “A beloved person never dies; he always lives on in our thoughts, words, and memories.” We extend our sincere gratitude for the joyful cooperation and meaningful scientific journey we shared with Prof. Philippe Jeandet – a man wholly devoted to science. He will forever remain in our hearts and minds, and we will always cherish the wonderful experience of working alongside him. We thank him for building bridges between scientists across the globe, for his boundless positive energy, and for showing that science transcends borders and serves as a force for good, freedom, and peace in the world. Once again, we express our deepest respect to Prof. Philippe Jeandet – as a scientist and as a human being. May his soul rest in eternal peace.
